# A dual colour fluorescence in situ hybridization (FISH) assay for identifying the zoonotic malaria parasite *Plasmodium knowlesi* with a potential application for the specific diagnosis of knowlesi malaria in peripheral-level laboratories of Southeast Asia

**DOI:** 10.1186/s13071-017-2273-7

**Published:** 2017-07-19

**Authors:** Jyotsna Shah, Akhila Poruri, Olivia Mark, Urmila Khadilka, Franziska Mohring, Robert W. Moon, Ranjan Ramasamy

**Affiliations:** 1 ID-FISH Technology, Palo Alto, CA USA; 2 IGeneX, Palo Alto, CA USA; 3 Kasturba Medical College Hospital, Mangalore, India; 4 London School of Hygiene and Tropical Medicine, Keppel Street, London, UK

**Keywords:** DNA probe, Fluorescence in situ hybridization, Malaria diagnosis, *Plasmodium knowlesi*, Zoonotic malaria

## Abstract

**Background:**

*Plasmodium knowlesi* is primarily responsible for zoonotic malaria in several Southeast Asian countries. Precise identification of the parasite in the blood of patients presently relies on an expensive and elaborate PCR procedure because microscopic examination of blood and other available field identification techniques lack adequate specificity. Therefore, the use of a simple and inexpensive dual-colour fluorescence in situ hybridization (FISH) assay, analogous to FISH assays recently described for *Plasmodium falciparum* and *Plasmodium vivax*, was investigated as a potential tool for identifying *P. knowlesi*.

**Results:**

A *P. knowlesi* 18S rDNA sequence-based DNA probe was used to test thin blood smears of *P. knowlesi* by FISH, and fluorescence viewed in a light microscope fitted with a light emitting diode light source and appropriate emission and barrier filters. The limit of detection in the *P. knowlesi* FISH assay was 84 parasites per μl in infected monkey blood and 61 parasites per μl for *P. knowlesi* cultured in human blood. The *P. knowlesi*-specific FISH probe detected only *P. knowlesi* and not *P. falciparum*, *Plasmodium malariae*, *Plasmodium ovale, P. vivax* or a panel of other human blood-borne pathogens. A previously described *Plasmodium* genus-specific probe used simultaneously in the *P. knowlesi* FISH assay reacted with all tested *Plasmodium* species.

**Conclusions:**

To our knowledge, this is the first description of a FISH assay for *P. knowlesi* that is potentially useful for diagnosing infections in remote laboratories in endemic countries.

**Electronic supplementary material:**

The online version of this article (doi:10.1186/s13071-017-2273-7) contains supplementary material, which is available to authorized users.

## Background

The four common species of human malaria parasites, i.e. *Plasmodium falciparum*, *Plasmodium vivax*, *Plasmodium malariae* and *Plasmodium ovale*, transmitted between humans by anopheline mosquito vectors, have an ancient zoonotic origin [1]. Zoonotic malaria is currently a relatively neglected aspect of human malaria. Alterations in patterns of human settlements in the vicinity of forests, and deforestation, are increasing human proximity to primate malaria hosts in the tropics causing zoonotic malaria to become an emerging human health concern [1]. Primate malaria parasites that are naturally transmitted by anopheline mosquito vectors to humans at the present time include the catarrhine monkey parasites *Plasmodium knowlesi* and *Plasmodium cynomolgi* in Southeast Asia, the platyrrhine monkey parasites *Plasmodium brasilianum* and *Plasmodium simium* in South America, and *Plasmodium vivax*-like parasites from apes in West Africa [1]. The clinical significance of *Plasmodium knowlesi* malaria is increasingly recognized in Southeast Asia after the initial discovery of a focus of human infections in the Kapit division of the Sarawak state in Malaysian Borneo [2], subsequently in peninsular Malaysia [3–5] and then in other Southeast Asian countries [6]. *Plasmodium knowlesi* is normally a parasite of *Macaca fascicularis* (the long-tailed or crab-eating macaque), *M. nemestrina* (the pig-tailed macaque), *Trachypithecus obscuras* (the dusky leaf monkey or spectacled langur) and *Presbytis melalophus* (the banded leaf monkey or brown langur) ([3, 6] and references therein). *Plasmodium knowlesi* infections in humans are commonly misidentified as *P. malariae* and *P. falciparum* during routine microscopic examination of Giemsa-stained blood smears because of morphological similarities between the blood stages [2–6]. Therefore, the definitive identification of human infections caused by *P. knowlesi* presently relies on PCR-based techniques [2–6]. The diagnosis of *P. knowlesi* malaria is important in a clinical context because its pathogenicity and drug treatment options can differ from the human malaria caused by *P. falciparum*, *P. malariae*, *P. ovale* and *P. vivax* that are also prevalent in the Southeast Asian region. Rapid immunochromatography-based diagnostic tests (RDTs) are being increasingly used worldwide to detect malaria but a reliable RDT for specifically detecting *P. knowlesi* is not yet available [7]. PCR-based assays are sensitive enough to detect 1–5 parasites per μl of blood and able to discriminate between the different *Plasmodium* species that infect humans, including *P. knowlesi* [8]. However PCR-based assays are relatively expensive, time consuming, and require specialized equipment. PCR-based techniques are therefore not suitable for routine diagnosis in typical peripheral and district-level diagnostic laboratories in the *P. knowlesi*-endemic regions of Southeast Asia.

Fluorescence in situ hybridization (FISH) is a cytogenetic technique using short complementary probes labeled with fluorescent molecules to localize and detect specific target DNA or RNA sequences. Because there are many copies of ribosomal RNA (rRNA) in the cytoplasm able to react with specific probes, rRNA can be visualized without amplification of the target sequence. It is also possible to find short nucleotide stretches that are unique to a genus or species making rRNA suitable for differentiating closely related pathogens [9, 10]. DNA probe-based FISH assays targeting 18S rRNA that detect *P. falciparum* and *P. vivax* in patient blood smears with appropriate diagnostic sensitivity and specificity, and suitable for use in peripheral laboratories in endemic areas, have recently been described [10]. The FISH assays can be performed with a light emitting diode (LED) light source with appropriate barrier and emission filters attached to a standard laboratory microscope [10]. To our knowledge, the development of an analogous FISH assay (termed the PK-FISH assay) capable of identifying *P. knowlesi* is described here for the first time.

## Methods

### Malaria parasites, other human pathogens and uninfected human blood

Thin smears of *P. knowlesi* on pre-cleaned microscope slides for FISH assays were prepared from two different sources of the parasite: (i) frozen blood from a West Malaysian strain of *P. knowlesi* first isolated in 1962 from *M. fascicularis* and subsequently maintained by serial passage in monkeys (ATCC 30192 obtained from American Type Culture Collection or ATCC, Atlanta, GA); and (ii) fresh in vitro cultures of the A1-H.1 clone, initially derived from the reference *P. knowlesi* H strain first isolated from a patient [11] and then recently adapted to grow in vitro in human red blood cells at the London School of Hygiene and Tropical Medicine, London, UK [12]. The *P. knowlesi* A1-H.1 strain parasites were grown in human blood obtained from the UK National Blood Transfusion Service using previously described culture conditions [12]. *Plasmodium knowlesi* cultures were synchronized by centrifugation through a cushion of Nycodenz (Axis-Shield, Oslo, Norway) as previously described [12].

*Plasmodium falciparum, P. malariae* and *P. ovale* blood smears from patients were provided by the Kenya Medical Research Institute/Walter Reed Project, Kisumu, Kenya, and *P. vivax* patient blood smears by the Kasturba Medical College Hospital, Mangalore, India, all being derived from anonymized left over patient samples as previously described [10]. In vitro cultures of *P. falciparum* strain 3D7 in human red blood cells were obtained from the London School of Hygiene and Tropical Medicine, London, UK.

Uninfected EDTA-treated human blood used as a control in the PK-FISH assay was obtained from ID-FISH Technology Inc., Palo Alto, CA. Bacterial and non-plasmodial protozoan blood-borne pathogens from different sources were used as additional specificity controls in the PK-FISH assay and these are detailed in Table 1.

**Table 1:** Reference bacterial and protozoan pathogens tested in the PK-FISH assay

Pathogen	Source
Bacteria	
*Anaplasma phagocytophilum*	Prof. Stephen J. Dumler, John Hopkins University, Baltimore, MA
*Borrelia burgdorferi*	ATCC 35210-B31
*Bartonella henselae*	ATCC 49882
*Ehrlichia chaffeensis*	Prof. Stephen J. Dumler, John Hopkins University, Baltimore, MA
*Leptospira interrogans*	ATCC 23476
Protozoa	
*Babesia microti*	ATCC 30221
*Babesia duncani*	ATCC PRA-302
*Trypanosoma cruzi*	Dr. George L. Stewart, University of West Florida, Pensacola, FL.
*Leishmania major* (amastigotes and promastigotes)	Kenya Medical Research Institute, Nairobi, Kenya

### FISH assay reagents

The *Plasmodium knowlesi* FISH assay kit (PK-FISH assay kit, catalogue number PkK04) used in the study was obtained from ID-FISH Technology Inc., Palo Alto, CA, USA. The kit contained *Plasmodium* genus- and *P. knowlesi*-specific DNA probes with hybridization buffer, smear preparation reagent (SPR), *Plasmodium* wash buffer, *Plasmodium* rinse buffer and *Plasmodium* mounting medium. The use of the *Plasmodium* genus-specific probe to detect the four common human *Plasmodium* species and also *P. knowlesi* by FISH has been previously described [10]. The *P. knowlesi*-specific probe in the PK-FISH assay kit (US patent application number 15/389,827) was based on a nucleotide sequence which has since been found to be conserved in all 18S rRNA sequences of different *P. knowlesi* isolates from humans and macaques deposited to date in GenBank. The PK-FISH assay was performed according to the manufacturer’s instructions provided with the kit.

In the PK-FISH assay kit, the *P. knowlesi*-specific probe was labeled with Alexa 488 green fluorescent dye and the *Plasmodium* genus-specific probe with Atto 550 orange fluorescent dye. Therefore with LED illumination, only *P. knowlesi* parasites are expected to appear green under a green filter (excitation 492 nm; emission 530 nm band pass) in the assay. Under an orange filter (excitation 560; emission 630 nm long pass), all *Plasmodium* species, including *P. knowlesi*, are expected to appear orange.

### Reactions of *P. knowlesi* and other blood-borne pathogens in the PK-FISH assay

Thin blood smears of *P. knowlesi* from monkey blood and in vitro culture in human blood, together with control smears from uninfected human blood, other human malaria parasite species derived from patients and the reference set of non-plasmodial blood-borne pathogens were tested in the PK-FISH assay to estimate its potential specificity for detecting *P. knowlesi*.

#### *Plasmodium knowlesi* from infected monkey blood

This was done first at the ID-FISH laboratories, Palo Alto, CA, USA, in order to establish the *P. knowlesi* FISH assay procedure. EDTA-treated monkey blood containing parasites was mixed with SPR at 3 parts blood: 1 part SPR by volume. A set of four thin smears was prepared from each test sample. Each smear was prepared from 4 μl of the mixture, air-dried and fixed in methanol for subsequent testing in the PK-FISH assay. Briefly, after addition of the two probes in 12 μl of hybridization buffer to each methanol-fixed smear, the smear was covered with a plastic cover-slip and placed in a humid chamber at 37 °C for 15 min for hybridization. After 15 min, each smear was washed twice for 2 min each with 1× *Plasmodium* wash buffer at ambient temperature, followed by a rinse with 1× *Plasmodium* rinse buffer. After drying the smears in complete darkness, a drop of *Plasmodium* mounting medium was added to each smear. The smear was then covered with a glass coverslip and viewed at ×1000 magnification, in an Olympus light microscope with an attached LED unit containing the filter set. A laboratory microscope with a LED light source and filter attachment is illustrated in Additional file 1: Figure S1.

#### *Plasmodium knowlesi* grown in vitro in human blood

A1-H.1 parasites from in vitro cultures in human red blood cells and synchronised as described to enrich for ring, trophozoite and schizont stages in different cultures [12] were used to prepare thin blood smears at the London School of Hygiene and Tropical Medicine and subsequently analyzed by the PK-FISH assay at the ID-FISH laboratories, Palo Alto, CA, USA, as described above for monkey blood.

#### Control *Plasmodium* species, uninfected human blood and other reference pathogens

Smears were prepared utilizing SPR treatment and then tested in the PK-FISH assay at the ID-FISH laboratories, Palo Alto, CA, USA, as described above for monkey blood. The presence of non-reacting cells in the test samples was confirmed by microscopy. Smears prepared from *Babesia duncani* grown in hamster blood (ATCC PRA-302) were also tested with a *Babesia* genus-specific FISH probe conjugated with Alexa 488 provided by the IGeneX clinical testing laboratory in Palo Alto, CA, USA to confirm the presence of *B. duncani* parasites.

### Limit of detection (LOD) of *P. knowlesi* in the PK-FISH assay

In a manner previously described for *P. falciparum* and *P. vivax* [10], the lowest concentration of parasites that could be detected in every one of the replicated smears at different serial dilutions of *P. knowlesi* from monkey blood or human blood culture was determined as the LOD.

#### *Plasmodium knowlesi* in monkey blood

The parasitemia determined by Giemsa staining was 42,000 parasites per μl in the original monkey blood sample. This was serially diluted in EDTA-treated normal human venous blood (ID-FISH laboratories, Palo Alto, CA, USA) at 1:5, 1:10, 1:50, 1:100, 1:500 and 1:1000 dilutions. At each dilution multiple thin blood smears were made and three smears tested in the PK-FISH assay. The concentration of parasites at the greatest dilution at which parasites could be detected by the *P. knowlesi*-specific probe in all three replicate smears was considered to be the provisional LOD. Once a provisional LOD was determined, an additional 17 smears were tested at the same dilution for confirming the LOD.

#### *Plasmodium knowlesi* grown in vitro in human blood

A1-H.1 culture at 3.9% parasitemia was diluted 1:10, 1:100 and subsequently at serial two-fold dilutions in EDTA-treated normal human blood at the London School of Hygiene and Tropical Medicine, London, UK, to prepare smears for determining the LOD. Initially two smears at 1:200 and greater dilutions were tested to determine a provisional LOD where both duplicate smears were positive in the PK-FISH assay. Based on the provisional LOD, 20 replicate smears at the same dilution as well as two-fold higher and two-fold lower dilutions were made and transported for testing by the PK-FISH assay in the ID-FISH laboratories, Palo Alto, CA, USA. The concentration of parasites at the greatest dilution at which all 20 replicate smears were positive in the assay was considered to be the LOD.

### Characteristics of the PK-FISH assay on blood smears containing both *P. falciparum* and *P. knowlesi*

To determine whether the concomitant presence of *P. falciparum* affects the detection of *P. knowlesi* due to a possible cross-reaction of the *P. knowlesi*-specific FISH probe with *P. falciparum*, the PK-FISH assay was performed on serial dilutions of *P. knowlesi* containing a relatively high concentration of *P. falciparum*. In vitro cultures of *P. falciparum* 3D7 and *P. knowlesi* A1-H.1 at 4% parasitaemia were used as starting material for this purpose at the London School of Hygiene and Tropical Medicine, London, UK. Beginning at a 1:400 dilution in uninfected human red blood cells estimated to yield 500 *P. knowlesi* per μl, the *P. knowlesi* culture was serially diluted two-fold up to a dilution of 1:25,600 in a stock of 1:400 dilution of the original *P. falciparum* culture in fresh uninfected human red blood cells estimated to contain 500 *P. falciparum* per μl. A 1:1 v/v mixture of the 1:400 dilutions of the *P. knowlesi* and *P. falciparum* cultures at 4% parasitaemia was also prepared. Thin blood smears were then made from the mixed parasites at the London School of Hygiene and Tropical Medicine, London, UK, and triplicate blood smears at each dilution of *P. knowlesi* were tested by the PK-FISH assay at the ID-FISH laboratories, Palo Alto, CA, USA, using the procedures outlined above.

## Results

### Reactions of *P. knowlesi* and other human malaria parasites and pathogens in the PK-FISH assay

**Fig. 1:**
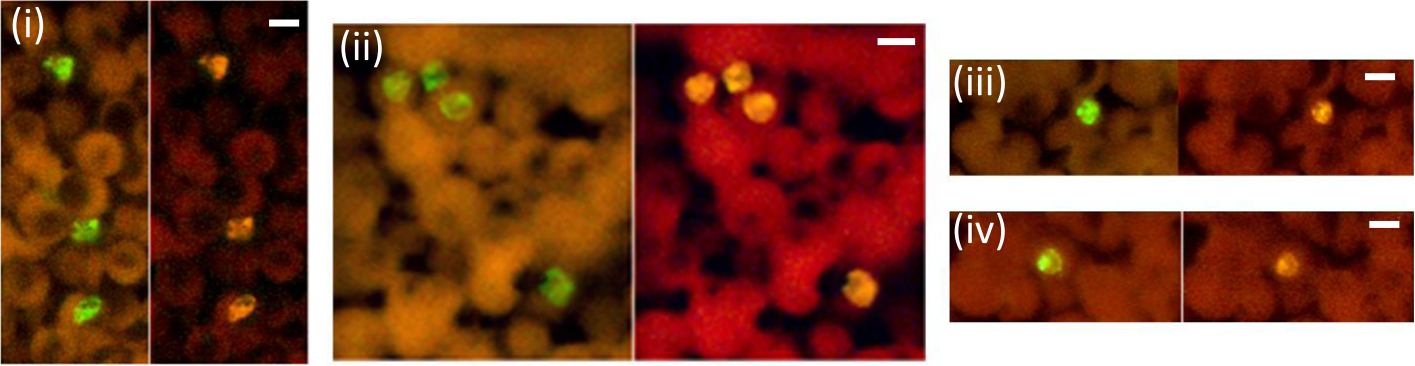
Fluorescence observed in the PK-FISH assay with the *P. knowlesi*-specific probe (*green*) and the *Plasmodium* genus-specific probe (*orange*) on monkey blood smears containing *P. knowlesi*. Dual colour fluorescence seen in a single microscope field at ×1000 magnification using the two different filters is shown in each set of paired photographs. The four sets of paired photographs are from four different fields (*i*) - (*iv*). *Scale-bars*: 5 μm

**Fig. 2:**
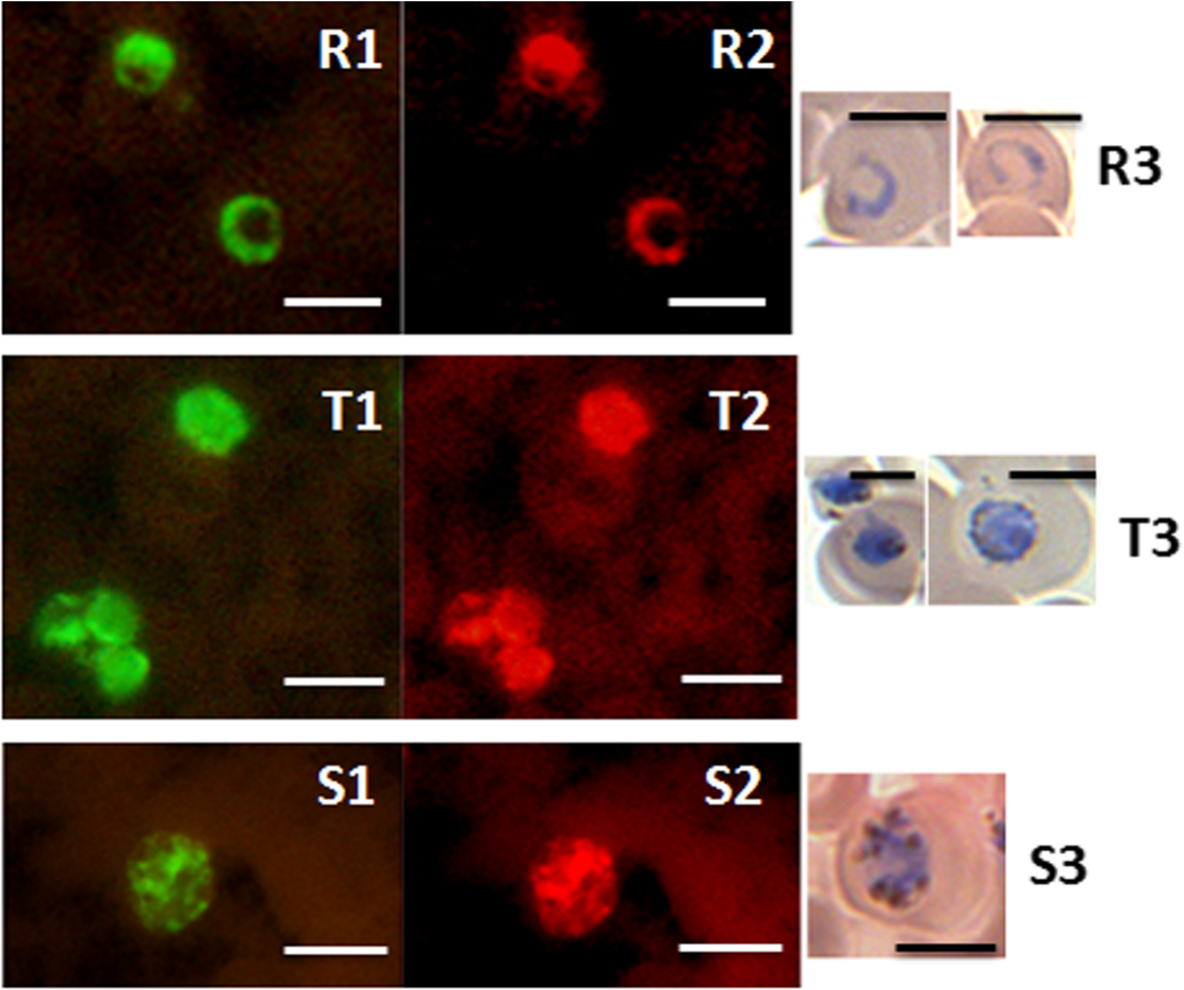
Fluorescence observed in the PK-FISH assay with the *P. knowlesi*-specific probe (*green*) and the *Plasmodium* genus-specific probe (*orange*) on different asexual stages of *P. knowlesi* A1- H.1 cultured in human blood. Dual colour fluorescence seen in the same microscope field at ×1000 magnification using the two different filters is shown in paired photographs R1 and R2, T1 and T2, and S1 and S2. Photographs of ring, trophozoite and schizont-stage parasites stained with Giemsa from smears prepared in parallel to the corresponding smears used in the PK-FISH assay are shown in R3, T3 and S3, respectively. *Scale-bars*: 5 μm. *Abbreviations*: R, rings; T, trophozoites; S, schizonts

**Fig. 3:**
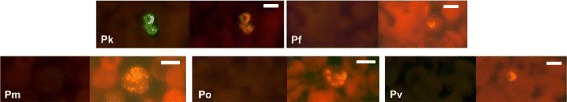
Fluorescence observed in the PK-FISH assay with the *P. knowlesi*-specific probe (*green*) and the *Plasmodium* genus-specific probe (*orange*) in blood smears containing *Plasmodium* spp. from monkey blood. Each set of paired photographs show the dual colour fluorescence in the same microscope field at ×1000 magnification using the two different filters. *Scale-bars*: 5 μm. *Abbreviations*: Pk, *P. knowlesi*; Pf, *P. falciparum*; Pm, *P. malariae*; Po, *P. ovale*; Pv, *P. vivax*

Use of the dual fluorescence *Plasmodium* genus-specific and the *P. knowlesi*-specific probes in the PK-FISH assay showed that both probes reacted simultaneously with *P. knowlesi* present in the same microscopic field of monkey blood (Fig. 1). Similarly, both probes could also label *P. knowlesi* A1-H.1 strain parasites, grown in human red blood cells in vitro (Fig. 2). By using synchronized in vitro cultures of *the* A1-H.1 enriched for either ring, trophozoite or schizont-stage parasites, we were able to demonstrate reactivity of the *P. knowlesi* and *Plasmodium* genus-specific probes against each stage of asexual development in the blood, with distinct staining patterns discernable for each parasite stage (Fig. 2). Additional tests showed that the *P. knowlesi*-specific probe used in the PK-FISH assay did not react with patient-derived *P. falciparum*, *P. vivax*, *P. malariae* or *P. ovale* that were however detected by the *Plasmodium* genus-specific probe in the same microscope field (Fig. 3).

**Fig. 4:**
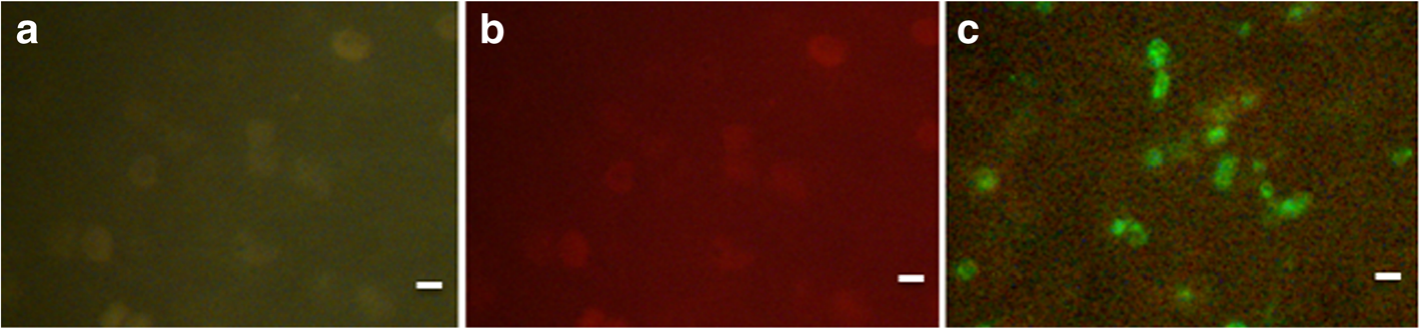
Fluorescence observed in the PK-FISH assay with the *P. knowlesi*-specific probe (*green*), the *Plasmodium* genus-specific probe (*orange*) and the *Babesia* genus-specific probe (*green*) in specificity control blood smears prepared from hamster blood infected with *B. duncani*. Two smears (Smear 1 and Smear 2) were made with the same *B. duncani*-infected hamster blood (ATCC PRA-302). Smear 1 was tested in the PK- FISH Assay and Smear 2 was tested in a *Babesia* genus-specific FISH assay. The results viewed at ×1000 magnification show **a** absence of a reaction in the PK-FISH assay with the *P. knowlesi*-specific probe on Smear 1, **b** absence of a reaction in the PK-FISH assay in the same microscope field with the *Plasmodium* genus-specific probe on Smear 1, and **c** reaction with the *Babesia* genus-specific FISH probe on Smear 2. *Scale-bars*: 5 μm

None of the other blood-borne bacterial and protozoan pathogens listed in Table 1 or uninfected human blood cells gave a positive reaction with either of the two probes in the PK-FISH assay. An example of non-reactive labelling is illustrated by the PK-FISH assay performed on *B. duncani*, another apicomplexan parasite closely related to the *Plasmodium* genus (Fig. 4). This shows that both the *P. knowlesi*-specific probe and the *Plasmodium* genus-specific probe fail to react with *B. duncani*. The *B. duncani* in the same hamster blood sample was however able to react with a *Babesia* genus-specific FISH probe (Fig. 4).

### Limit of detection of *P. knowlesi* in the PK-FISH assay

The greatest dilution at which the monkey blood-derived *P. knowlesi* was detected in the PK-FISH assay in every one of the 20 replicate smears was 1:500. Based on the original parasitemia of 42,000 parasites per μl determined by Giemsa staining, this corresponded to a LOD of 84 *P. knowlesi* parasites per μl of blood.

The greatest dilution of the culture at which the in vitro grown *P. knowlesi* A1-H.1 could be detected in both duplicate smears in the PK-FISH assay was 1:6400. This corresponds to a provisional LOD of 61 parasites per μl of blood. The LOD was confirmed with the 20 additional smears tested at 1:6400 dilution as all smears were positive at this dilution. At a 1:3200 dilution corresponding to 123 parasites per μl, all 20 replicate smears were also positive in the PK-FISH assay and this was also the case with the duplicate smears initially tested at this and lower dilutions. At 1:12,800 dilution corresponding to 31 parasites per μl, 16 of the 20 replicate smears were positive giving a detection rate of 80%. At 1:25,600 and greater dilutions of the culture, parasites could not be detected in either of the duplicate smears by the PK-FISH assay.

The LOD of *P. knowlesi* with the *Plasmodium* genus-specific probe observed under orange filter was the same as that observed with the *P. knowlesi*-specific probe under the green filter in both monkey blood and in vitro human red blood cell culture.

### PK-FISH assay on mixed preparations of *P. falciparum* and *P. knowlesi*

**Fig. 5:**
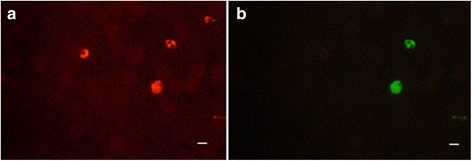
The reaction of a mixture of *P. falciparum* and *P. knowlesi* in the PK-FISH assay. In vitro cultures of *P. falciparum* and *P. knowlesi* were mixed 1:1 *v*/v to yield 250 parasites per μl of each parasite and then tested in the PK-FISH assay. The dual colour fluorescence observed in the same field viewed at ×1000 magnification with **a** the *Plasmodium* genus-specific probe (*orange*) and **b** the *P. knowlesi*-specific probe (*green*) are shown. *Scale-bars*: 5 μm

Mixed infections of multiple species of malaria parasite in a single patient are common and therefore we additionally tested the ability of the PK-FISH assay to detect *P. knowlesi* in the context of a mixed infection. For this, we artificially created mixed infections using in vitro cultured *P. knowlesi* and *P. falciparum* for the assay. The lowest concentration of *P. knowlesi* that could be detected in the presence of *P. falciparum* by the PK-FISH assay in all three replicate smears was 16 *P. knowlesi* parasites per μl. This was achieved in the concomitant presence of *P. falciparum* at approximately 500 parasites per μl. The absence of a reaction of the *P. knowlesi*-specific probe with *P. falciparum* in smears containing a mixture of *P. knowlesi* and *P. falciparum* at a final concentration of 250 parasite per μl each in the PK-FISH assay is illustrated in Fig. 5, where the *Plasmodium* genus-specific probe is concurrently shown to react with both *P. falciparum* and *P. knowlesi.*

## Discussion

The FISH assays developed for *P. falciparum* and *P. vivax* were reported to be at least as sensitive and more specific than microscopic examination of Giemsa-stained slides, and directly applicable for diagnosis in malaria-endemic countries [10]. The desirable characteristics of these FISH assays were attributed to the selection of suitable probe sequences, appropriate smear preparation to facilitate penetration of DNA probes through the cell membrane and good hybridization properties. The present findings suggest that a similar FISH assay for *P. knowlesi*, described here for the first time can accurately identify the parasite, potentially overcoming the problem of misidentifying *P. knowlesi* as *P. malariae* or *P. falciparum* in Southeast Asia. The *P. knowlesi*-specific and the *Plasmodium* genus-specific probes in the PK-FISH assay were 100% accurate in detecting the two different *P. knowlesi* isolates tested and the 13 reference pathogens. The LOD of parasites in the PK-FISH assay was at least 84 *P. knowlesi* parasites per μl in thin blood smears which is comparable to routine microscopy with Giemsa-stained blood films [10]. The LOD of similar FISH assays for *P. falciparum* and *P. vivax* were reported to be 55 and 59 parasites per μl respectively [10]. The LOD may be improved if the FISH assay can be adapted to thick blood smears but our observations suggest that this is not yet possible. The PK-FISH assay is however likely to be equally effective on smears made directly from finger prick samples of patient blood as shown previously for the analogous *P. falciparum*- and *P. vivax*-specific FISH assays [10]. Because RNA is rapidly degraded in dying cells, the FISH assays preferentially detect live parasites in patient blood as previously reported [10]. Therefore the PK-FISH assay may also be useful for monitoring the in vivo response of *P. knowlesi* to drug treatment.

The PK-FISH assay described here has the added advantage of being potentially able to simultaneously detect mixed infections of *P. knowlesi* and other *Plasmodium* species seen in some patients [2, 5]. This is because the other *Plasmodium* species will bind the *Plasmodium* genus-specific probe but not the *P. knowlesi*-specific probe and can subsequently be identified by other species-specific FISH assays [10]. There was no evidence for a cross-reaction of the *P. knowlesi*-specific probe with *P. falciparum* when it was tested with a mixture of the two parasites derived from in vitro cultures because there was no indication that the LOD for *P. knowlesi* was reduced and no fluorescence reaction was observed with the probe on *P. falciparum*.

There is evidence for at least two genetically dimorphic *P. knowlesi* populations in Southeast Asia, likely to be associated with adaptation to the two main macaque hosts, *M. fascicularis* and *M. nemestrina* [13–16]. The rRNA sequences available in GenBank, which include reported sequences from the two populations [15], suggest that the specifically selected target rRNA sequence for the *P. knowlesi*-specific probe used here does not differ between the two populations. This can be further investigated with knowlesi malaria patients in Southeast Asia. Blood samples or smears from patients with *P. knowlesi* malaria in endemic areas of Southeast Asia were however not available for testing in the PK-FISH assay during the present investigation. Studies with a large number of such blood samples are necessary to determine the clinical diagnostic parameters and applicability of the PK-FISH assay in knowlesi malaria-endemic countries.

Another technique that rapidly detects *P. knowlesi* infections in human blood uses the loop-mediated isothermal amplification (LAMP) procedure, but this has not yet been adapted for routine use in endemic areas [17]. The advantages of FISH assays for malaria compared to the more sensitive nested PCR assays have been previously discussed in detail for the *P. falciparum* and *P. vivax* FISH assays [10], and the same considerations also apply to the PK-FISH assay. In essence, the FISH assays require no specialized equipment other than a light microscope with a LED source and filter attachments, and are less costly and simpler to perform than PCR-based tests. A single LED and filter attachment on a microscope may also be used for several different pathogen-specific FISH assays. The LED can be powered by low voltage batteries in areas where the mains electricity supply is not reliable and offers many other advantages over mercury arc lamps used in conventional fluorescence microscopes [10]. Another important consideration for delivery and use in tropical countries is that the PK-FISH assay kit is stable at 30 °C for several months. The PK-FISH assay is therefore potentially useful for diagnosing *P. knowlesi* malaria in peripheral and district-level diagnostic laboratories in Southeast Asian countries. It also has similar advantages in this respect to the dual colour fluorescence FISH assays recently described for identifying *Mycobacterium tuberculosis* and *Mycobacterium avium* complexes [18].

The need for a simple and rapid assay for diagnosing *P. knowlesi* in peripheral and district-level endemic area-laboratories is illustrated by two recent case reports of knowlesi malaria in (i) a person working in a mining development in Indonesian Borneo [19] and (ii) a Sri Lankan soldier returning from training in peninsular Malaysia [20]. PCR-based diagnosis was the only available procedure for specific diagnosis in both cases and this involved recourse to a central molecular biology facility, in Jakarta in the Indonesian case and Singapore in the Sri Lankan case [19, 20].

## Conclusions

The PK-FISH assay described here has the potential for meeting an important diagnostic need in peripheral and district-level clinical laboratories in *P. knowlesi*-endemic areas. Investigations with pertinent patient blood samples in Southeast Asia are however needed to establish its value in the clinical diagnosis of knowlesi malaria.

## Additional file

Additional file 1:Figure S1. Photograph showing a laboratory microscope fitted with a LED and filter attachment for viewing fluorescence in FISH assays (reproduced with permission from [18]. (PPTX 70 kb)

## Abbreviations

ATCC: American type culture collection
EDTA: Ethylenediaminetetraacetic acid
FISH: Fluorescence in situ hybridization
LAMP: Loop mediated isothermal amplification
LED: Light emitting diode
LOD: Limit of detection
PCR: Polymerase chain reaction
PK-FISH: *Plasmodium knowlesi* fluorescence in situ hybridization
rRNA: ribosomal RNA
SPR: Smear preparation reagent
