# Supplementary figures and images for: A dual colour fluorescence in situ hybridization (FISH) assay for identifying the zoonotic malaria parasite Plasmodium knowlesi with a potential application for the specific diagnosis of knowlesi malaria in peripheral-level laboratories of Southeast Asia
Source: Parasit Vectors. 2017 Jul 19;10:342. doi: 10.1186/s13071-017-2273-7 (PMC5517825; doi:10.1186/s13071-017-2273-7)

## Slide 1
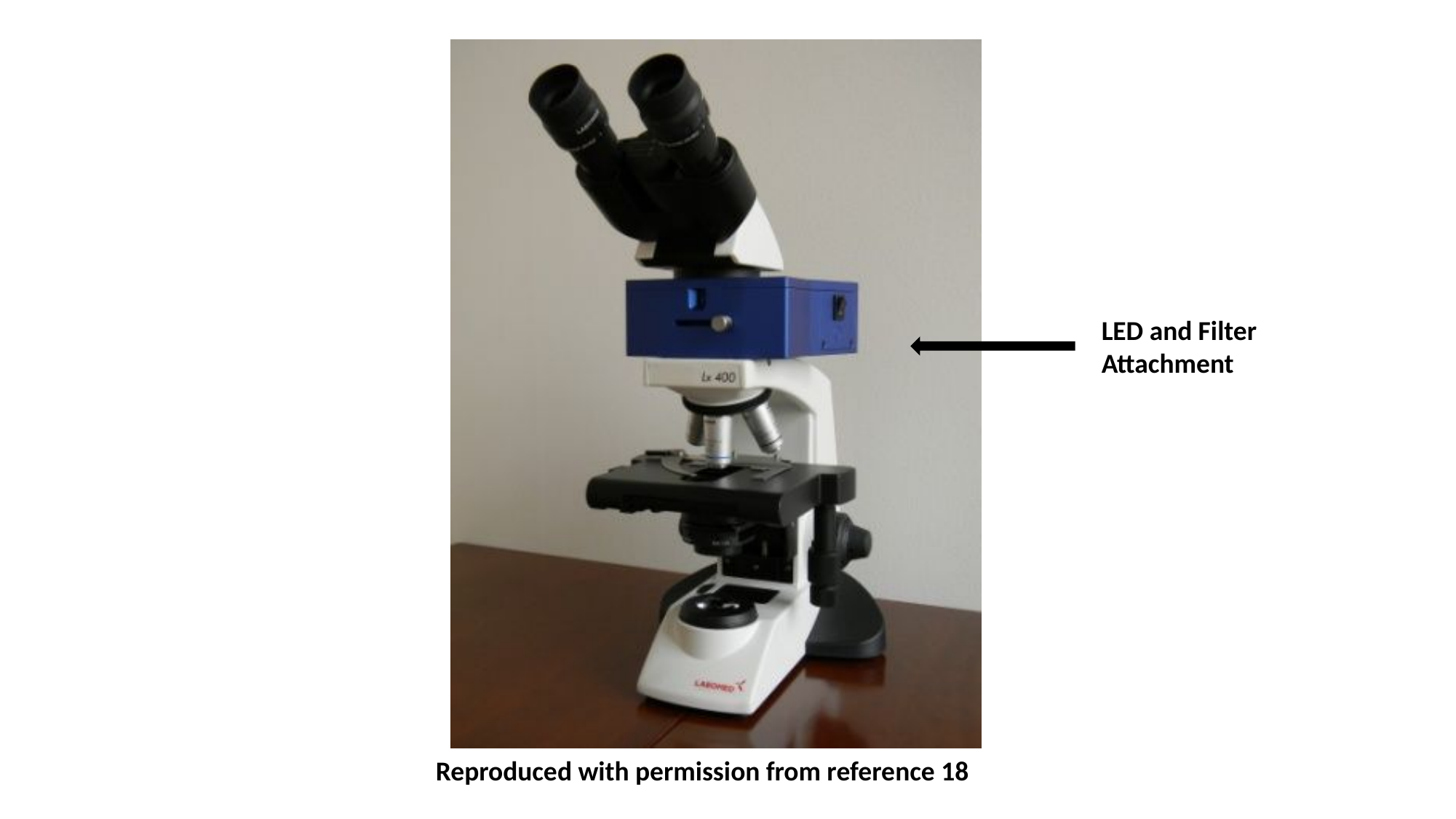

LED and Filter
Attachment
 Reproduced with permission from reference 18

Supplement: Additional file 1:Figure S1. — Photograph showing a laboratory microscope fitted with a LED and filter attachment for viewing fluorescence in FISH assays (reproduced with permission from [18]. (PPTX 70 kb) [file 13071_2017_2273_MOESM1_ESM.pptx]
